# Identification of a Mitochondria-Related Gene Signature to Predict the Prognosis in AML

**DOI:** 10.3389/fonc.2022.823831

**Published:** 2022-03-10

**Authors:** Nan Jiang, Xinzhuo Zhang, Qi Chen, Fahsai Kantawong, Shengli Wan, Jian Liu, Hua Li, Jie Zhou, Bin Lu, Jianming Wu

**Affiliations:** ^1^ School of Pharmacy, Southwest Medical University, Luzhou, China; ^2^ Foreign Language School, Southwest Medical University, Luzhou, China; ^3^ Faculty of Associated Medical Sciences, Chiang Mai University, Chiang Mai, Thailand; ^4^ The Affiliated Hospital of Southwest Medical University, Southwest Medical University, Luzhou, China; ^5^ The Second Affiliated Hospital, Hengyang Medical School, University of South China, Hengyang, China

**Keywords:** acute myeloid leukemia, prognostic signature, mitochondria-related genes, The Cancer Genome Atlas, Therapeutically Applicable Research to Generate Effective Treatments, LASSOR

## Abstract

Mitochondria-related metabolic reprogramming plays a major role in the occurrence, development, drug resistance, and recurrence of acute myeloid leukemia (AML). However, the roles of mitochondria-related genes (MRGs) in the prognosis and immune microenvironment for AML patients remain largely unknown. In this study, by least absolute shrinkage and selection operator (LASSO) Cox regression analysis, 4 MRGs’ (HPDL, CPT1A, IDH3A, and ETFB) signature was established that demonstrated good robustness in TARGET AML datasets. The univariate and multivariate Cox regression analyses both demonstrated that the MRG signature was a robust independent prognostic factor in overall survival prediction with high accuracy for AML patients. Based on the risk score calculated by the signature, samples were divided into high- and low-risk groups. Gene set enrichment analysis (GSEA) suggested that the MRG signature is involved in the immune-related pathways. *Via* immune infiltration analysis and immunosuppressive genes analysis, we found that MRG risk of AML patients was strikingly positively correlated with an immune cell infiltration and expression of critical immune checkpoints, indicating that the poor prognosis might be caused by immunosuppressive tumor microenvironment (TME). In summary, the signature based on MRGs could act as an independent risk factor for predicting the clinical prognosis of AML and could also reflect an association with the immunosuppressive microenvironment, providing a novel method for AML metabolic and immune therapy based on the regulation of mitochondrial function.

## Introduction

Acute myeloid leukemia (AML) is a common hematological cancer, characterized by the accumulation of undifferentiated myeloid progenitor in the hematopoietic system, leading to normal blood component decrease, severe infections, anemia, and hemorrhage (1). AML patients’ genomes carry the fewest mutations discovered in most other cancers, with about 13 coding mutations found per patient (2). Accumulating research reported potential driver mutation and epigenetic abnormalities related to AML pathogenesis; however, the therapeutic strategy for AML patients has remained chemotherapy with or without stem cell transplantation for many years (2–4). Despite advanced progress in early diagnosis, drug mining, and multidisciplinary tumor management, the long-term overall survival (OS) of AML patients remains poor (5–7). Therefore, it is urgent to identify novel and effective potential biomarkers and prognostic models to improve treatment allocation by identifying patients at high risk of a poor prognosis.

Mitochondria are at the center of energy production and are important for cell growth, proliferation, differentiation, and death (8). Therefore, mitochondria are fundamentally involved in cancer-related biological processes, including cancer initiation, development, invasion, recurrence, and drug resistance (9). Many studies reported that epigenetic modulation and mutation of mitochondria-related genes (MRGs) and bio-energetic reprogramming are important in cancer pathogenesis (9, 10). Moreover, studies have found that the mitochondria-related biology process is a potential cancer therapy (11, 12). Recent studies have reported that AML cells have a dependency on mitochondrial function, especially leukemia stem cells. Targeting mitochondrial respiration became a novel treatment of AML (13). Thus, exploration of underlying mitochondria-related alterations in AML patients may bring out some novel insights to promote the prognosis.

In this study, by differential expression analysis, univariate Cox regression, and 10-fold least absolute shrinkage and selection operator (LASSO) Cox regression analysis, an MRG signature was established to predict the prognosis of AML patients. Gene set enrichment analysis (GSEA) has been performed to explore the functional change in the high-risk group. Single-sample GSEA (ssGSEA) immune infiltration analysis and immunosuppressive genes analysis were applied to investigate immune cell infiltration and immunosuppressive condition of AML. In summary, our results demonstrate that the signature based on MRGs could act as a reliable independent biomarker for predicting the clinical prognosis of AML, and high MRG risk AML patients were closely associated with an immunosuppressive microenvironment. Therefore, our study may provide a novel method for AML metabolic and immune therapy based on the regulation of mitochondrial function.

## Materials and Methods

### Data Acquisition

RNA-seq data and clinical data (149 AML samples) from TCGA-AML cohorts combined with whole blood cohorts (337 normal whole blood samples) from GTEx were downloaded from the UCSC Xena database (https://xenabrowser.net/datapages/). We also obtained clinical and expression data of AML patients from the Therapeutically Applicable Research to Generate Effective Treatments (TARGET) database (https://ocg.cancer.gov/programs/target) as the validation set to validate our prognostic model. All eligible samples from The Cancer Genome Atlas (TCGA) and validation sets were collected according to the following inclusive criteria: 1) diagnosed AML specimen; 2) availability of transcriptome data; and 3) availability of general survival information and related clinical data. The corresponding information of AML samples is shown in **Table 1**.

**Table 1:** Clinical information of samples.

Clinical features	TCGA-AML dataset (n = 149)		p-Value	Clinical Features	TARGET-AML dataset (n = 187)		p-Value
	High-risk group	Low-risk group			High-risk group	Low-risk group	
Vital status			0.004	Vital status			0.002
Alive	19	37		Alive	35	56	
Dead	55	38		Dead	59	37	
Gender			0.363	Gender			0.943
female	31	38		Female	49	47	
male	43	37		Male	45	46	
BM blast			0.211	BM blast			0.136
<70%	63	55		<70%	30	40	
≥70%	12	19		≥70%	62	50	
Age class			0.036	Age class			0.947
<55	24	38		<10	53	51	
≥55	50	37		≥10	41	42	
FAB category			<0.001	FAB category			<0.001
M0	5	8		M0	12	11	
M1	16	17		M1	10	11	
M2	13	22		M2	15	30	
M3	1	12		M3	0	0	
M4	19	14		M4	23	29	
M5	16	1		M5	30	5	
M6	2	0		M6	2	1	
M7	2	1		M7	2	6	

BM, bone marrow.

### Identification of Differentially Expressed Mitochondria-Related Genes

MRGs in the present study were defined as the coding genes of mitochondria-located proteins, including all proteins located in the mitochondrial membrane, matrix, cristae, and mitochondria-associated endoplasmic reticulum membranes. Depending on subcellular localization, a total of 1,136 mitochondria-located genes were downloaded from MitoCarta3.0 (14) (https://www.broadinstitute.org/) (**Supplementary Table 1**). Then, we extracted the MRG expression data from TCGA-GTEx gene expression dataset (337 normal blood samples and 149 AML samples). All the RNA-seq data have been pre-normalized by GDC mRNA analysis pipeline. The differentially expressed MRGs (DE MRGs) between the AML samples and normal controls were identified using the combination of DESeq2, EdgeR, and Limma (voom). Log2 |Fold Change| > 1 and adjusted p < 0.05 were used as the cutoff to screen DE MRGs.

### Bio-Functional Analysis of the Acute Myeloid Leukemia-Related Mitochondria-Related Genes

The bio-functional enrichment analysis of DE MRGs, including Gene Ontology (GO) analysis and Kyoto Encyclopedia of Genes and Genomes (KEGG) pathway enrichment analyses, was conducted using the clusterProfiler and enrichplot packages (15, 16), using MRGs as background genes and p < 0.05 as the cutoff. To find out whether bio-function differed between low- and high-risk patients, we also performed KEGG pathways and AML-related GSEA using the GSEA software (GSEA 4.0.3) (17, 18). For each analysis, the permutations of the gene set were all performed 1,000 times.

### Establishment of Prognostic Classifiers

A univariate Cox regression was performed for all DE MRGs, and the genes with p < 0.05 were identified as prognostic MRGs. Then the 10-fold LASSO cross-validation Cox regression analysis was applied to all prognostic MRGs for selection of the most useful biomarkers and to build a survival predicting classifier. LASSO is a popular prognostic model-building method of compression estimation, which can automatically remove unnecessary features and only keep the most important variables in the final model (19). The predicting risk scores were calculated based on the following formula:


Risk Score=Σ(Cox coefficient×Genes expression levels)

The low- and high-risk groups of AML patients were divided by the median risk score. The predictive ability of the model for training and validation cohorts was evaluated using the receiver operating characteristic (ROC) curve analysis, Kaplan–Meier (KM) log-rank test, and univariate and multivariate Cox regression analyses.

### Estimation of Immune Cell Type Proportion

In order to further study the relationship between model predicting risk and immune cell infiltration, the ssGSEA algorithm was used to estimate the abundances of 28 immune cell types obtained from Charoentong et al. (20). To avoid the blast cell signal from overwhelming the immune environment cell signals, we extract samples with <70% blast for the immune environment analysis. The abundance of immune cells was calculated according to the expression of the reference gene within the gene set from transcriptomic data, using the GSVA package *via* R software (21).

### Statistical Analysis

All statistical analysis and figure construction were conducted by R version 4.1 (http://www.R-project.org) and GraphPad Prism 8.0 statistical software (GraphPad Software, Inc., La Jolla, CA, USA). The KM survival analysis was performed and analyzed using a log-rank test. The correlation between risk score and clinicopathological characteristics was analyzed by the chi-square test. The Wilcoxon test was used to compare the difference between groups. In all the statistical analyses, p-value <0.05 was considered statistically significant.

## Results

### The Differential Expression of Mitochondria-Related Genes in Acute Myeloid Leukemia

**Figure 1:**
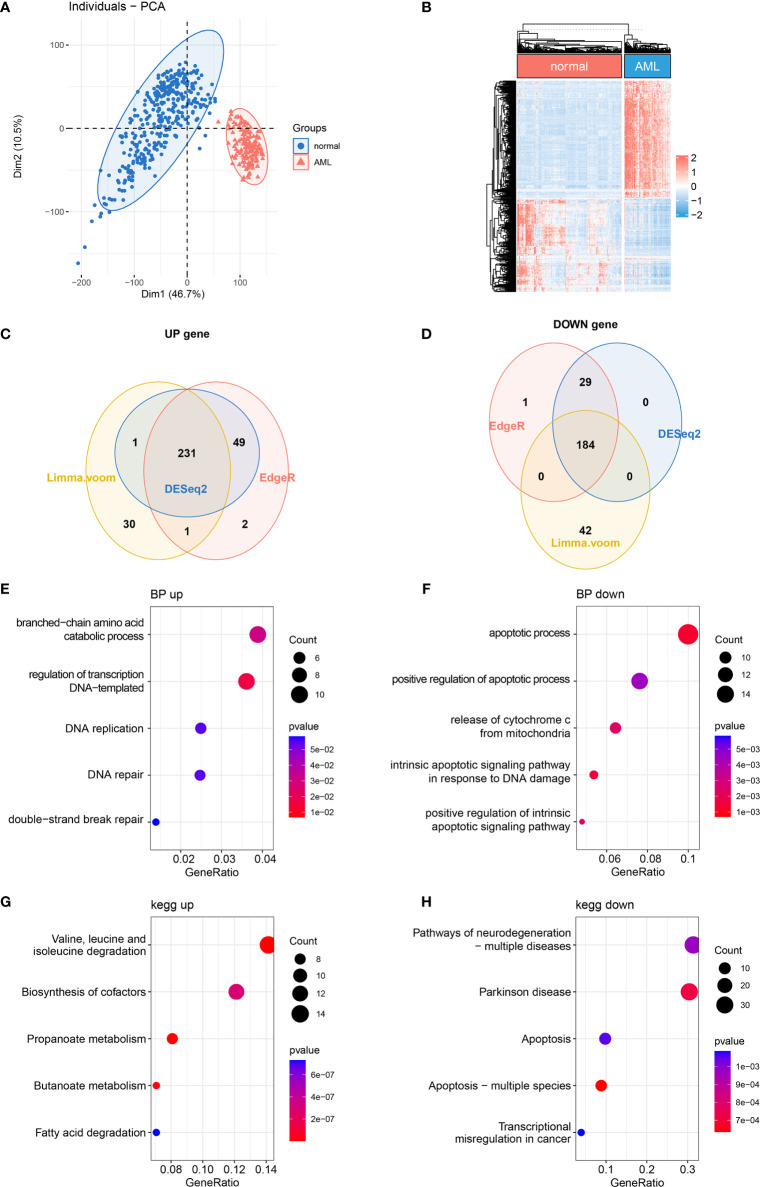
Differentially expressed MRG analysis. **(A)** PCA and **(B)** DE MRG heatmap. **(C, D)** Venn plots of 3 different DE analyses. **(E, F)** Bubble plots of GO enrichment analysis of DE MRGs. BP, biological process. **(G, H)** Bubble plots of KEGG pathway enrichment analysis of DE MRGs. MRG, mitochondria-related gene; PCA, principal component analysis; DE MRGs, differentially expressed MRGs; GO, Gene Ontology; KEGG, Kyoto Encyclopedia of Genes and Genomes.

It has been reported that mitochondrial alterations led to metabolic vulnerabilities in AML cells and participated in AML development in various ways (22, 23). To find out the DE MRGs in AML, 149 AML samples and 337 normal whole blood samples were extracted from TCGA-GTEx datasets. First, principal component analysis (PCA) was performed to check the quality of the expression data, showing good separation between normal and AML groups (**Figure 1A**). Then, 3 differential expression analyses were performed to identify DE MRGs (**Supplementary Figures 1A** **–F**). A total of 415 common DE MRGs were identified, while 184 of these were upregulated and 231 were downregulated (**Figures 1B** **–D**). Detailed information on DE MRGs is given in **Supplementary Table 2**. GO and KEGG were performed to explore the biology function change related to mitochondria. GO biological process analysis showed that the upregulated MRGs were associated with the DNA replication and DNA repair, which was closely related to AML cell proliferation (**Figure 1E**), and the downregulated MRGs were associated with the apoptotic process and apoptosis pathways, which showed an anti-apoptosis ability in AML cells (**Figure 1F**). Moreover, according to KEGG enrichment analysis, upregulated MRGs were enriched in organic molecule degradation and metabolism (**Figure 1G**), and downregulated MRGs were also connected with apoptosis (**Figure 1H**). The functional enrichment analysis above suggested that AML was associated with promoting proliferation and anti-apoptosis.

### Construction of a Prognostic Models Composed of 4 Mitochondria-Related Genes’ Signature

**Figure 2:**
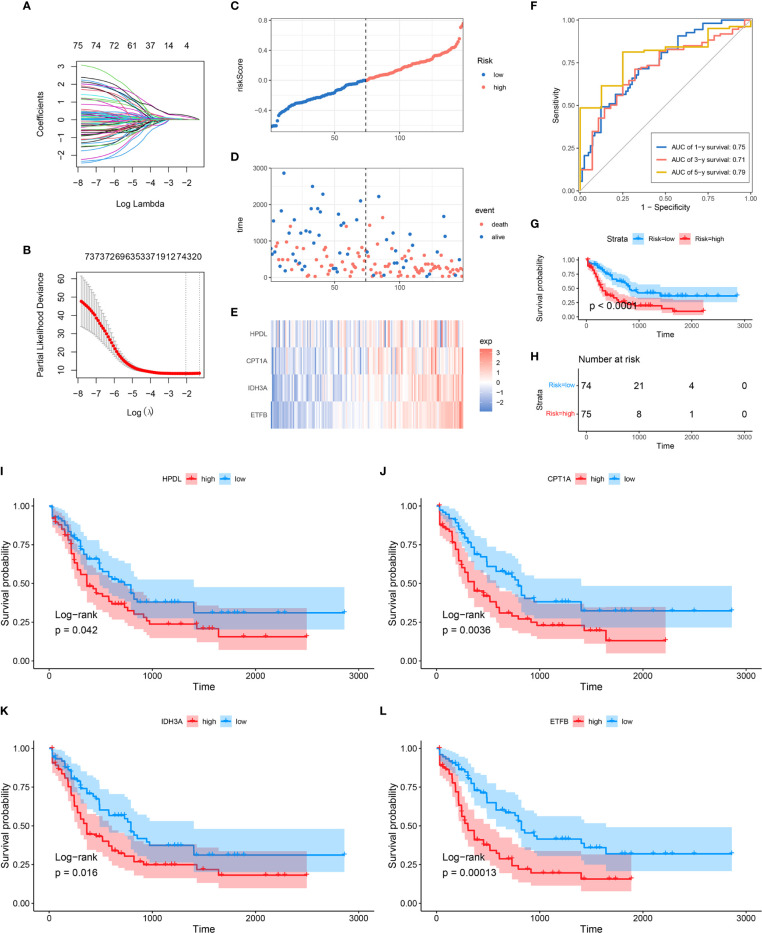
Construction of MRG-based prognostic classifier. **(A)** Each gene’s trajectory. The horizontal axis represents the log value of the gene lambda, and the vertical axis represents the independent gene’s coefficient. **(B)** CIs with different values of lambda, **(C–E)** Distribution of risk score, survival status, and expression of 4 MRGs in TCGA-AML cohort. **(F)** ROC curve of 4 MRGs’ signature prediction. **(G, H)** KM survival curves between two risk groups based on 8-gene signature classification. **(I–L)** The KM survival plots of 4 MRGs. MRG, mitochondria-related gene; ROC, receiver operating characteristic; KM, Kaplan–Meier.

In order to further explore the prognostic value of MRG in AML, univariate Cox regression analysis was performed to identify the clinically relevant MRGs from 415 differentially expressed genes (DEGs), and 76 of them were significantly associated with OS (**Supplementary Table 3**). To minimize model over-fitting, LASSO regression was applied to construct the prognostic model. Thus, the independent variable’s trajectory was explored in **Figure 2A**, and 10-fold cross-validation was used to analyze the CI under each lambda, as shown in **Figure 2B**. We finally established a mitochondria-related prognostic signature with 4 MRGs, including HPDL, CPT1A, IDH3A, and ETFB. The LASSO correlation coefficient of each MRG is shown in **Table 2**. Risk scores were calculated according to the expression level of the sample, and the risk score distribution is explored in **Figures 2C–E**, showing that the proportion of death with a high-risk score is significantly higher than that of samples with a low-risk score and expressions of 4 MRGs all upregulated as the risk score went up (**Supplementary Figure 2**). ROC was applied to evaluate the predictive classification efficiencies of the LASSO model, as shown in **Figure 2F**. The area under the curve (AUC) values of the model were 0.75, 0.71, and 0.79 at 1, 3, and 5 years. A KM plot was drawn using samples divided into the high- and low-risk groups by median risk score, showing the high-risk group had a poorer prognosis with significant difference (p < 0.0001) (**Figures 2G, H**). Finally, the KM plots of the 4 MRGs showed significantly predictive ability for the prognosis of patients in high- and low-expression groups (**Figures 2I** **–L**).

**Table 2:** The MRGs in the prognostic classifier.

Gene	Univariate Cox regression analysis			LASSO coefficient
	HR	95% CI	p-Value	
HPDL	1.43	1.11–1.83	0.0049	0.019734
CPT1A	1.69	1.26–2.26	5.00E−04	0.027628
IDH3A	1.79	1.35–2.38	1.00E−04	0.170339
ETFB	1.87	1.42–2.45	0	0.245829

MRGs, mitochondria-related genes; HR, hazard ratio; LASSO, least absolute shrinkage and selection operator.

### Robust Validation of Mitochondria-Related Gene Risk Signature in Different Cohorts

**Figure 3:**
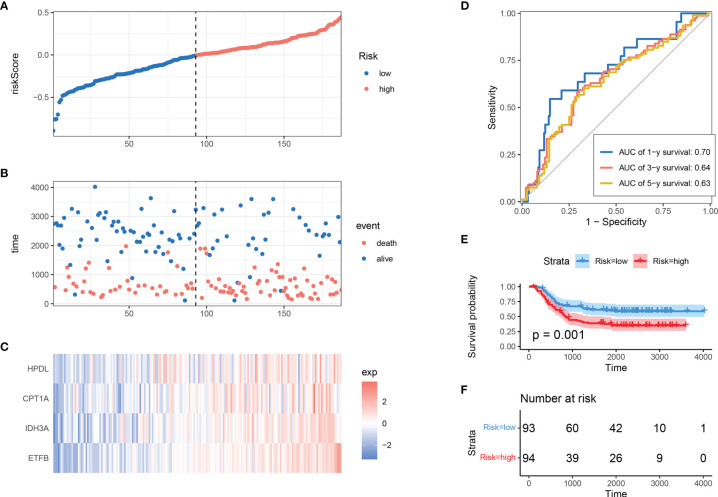
Validation of MRG-based prognostic classifier. **(A–C)** Distribution of risk score, survival status, and expression of 4 MRGs along with risk score in TARGET-AML cohort. **(D)** ROC curve of 4 MRGs’ signature classification in TARGET-AML cohort. **(E, F)** Survival curves between two risk groups based on 4 MRGs’ signature classification in TARGET-AML cohort. MRG, mitochondria-related gene; ROC, receiver operating characteristic.

To determine the model’s robustness, TARGET-AML datasets were introduced as an independent validation cohort. The risk score of each sample was calculated and explored according to the same LASSO coefficients and the expression level of 4 MRGs (**Figures 3A** **–C**). In accordance with TCGA training set, the samples with high-risk scores had a higher death proportion than those with low-risk scores. Moreover, in the TARGET-AML cohort, the ROC for 1, 3, and 5 years was 0.7, 0.64, and 0.63, respectively (**Figure 3D**). The KM plot also showed that the high-risk group had a poorer prognosis with significant difference (p = 0.001) (**Figures 3E, F**). The above results show that the MRG model had good robustness with prognostic predictive ability in different cohorts.

### Relationship Between Mitochondria-Related Genes’ Expression and Cytogenetic Risk in Acute Myeloid Leukemia

**Figure 4:**
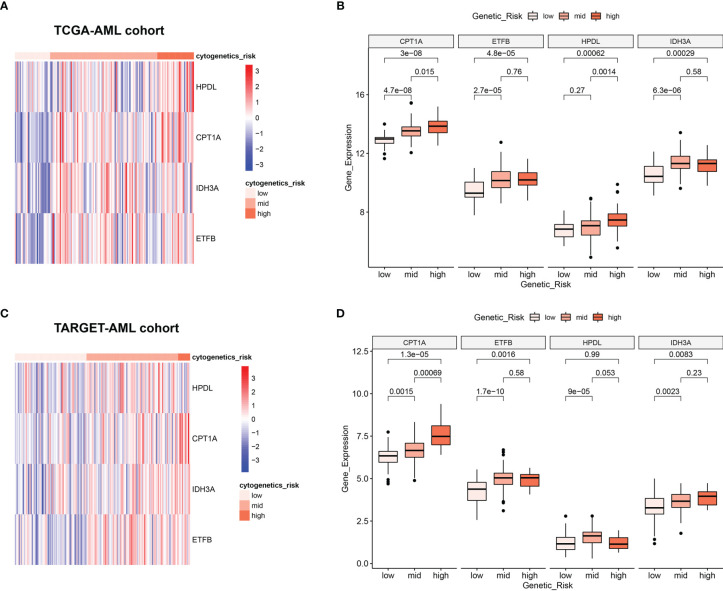
Relationship between 4 MRGs’ expression and cytogenetic risk in AML. **(A)** The heatmap of 4 MRGs in different risk stratification in TCGA-AML. **(B)** The boxplot of the 4 MRGs in different risk stratification in TCGA-AML. **(C)** The heatmap of 4 MRGs in different risk stratification in TARGET-AML. **(D)** The boxplot of the 4 MRGs in different risk stratification in TARGET-AML cohort. AML, acute myeloid leukemia.

Clinically, AML patients are often divided into different groups according to their clinical characteristics and morphology, immunology, cytogenetics and molecular biology (MICM) feature, which is known as cytogenetic risk stratification (24, 25). The relationship between AML cytogenetic risk stratification and the expression level of 4 MRGs was analyzed. The heatmap demonstrated 4 MRGs’ expression distribution along with AML cytogenetic risk stratification in TCGA cohort (**Figure 4A**), showing that the expression of 4 MRGs significantly went up in the higher cytogenetic risk group. Quantitative statistics further confirmed the significant difference of MRG expression levels in different risk stratification (**Figure 4B**), showing that the 4 MRGs’ expression levels went up along with the increased risk stratification. The expression trend of the 4 MRGs was further confirmed by the TARGET-AML cohort, showing the same pattern of the AML patients in TCGA (**Figures 4C, D**).

### Clinical Independence of Mitochondria-Related Gene Signature

**Figure 5:**
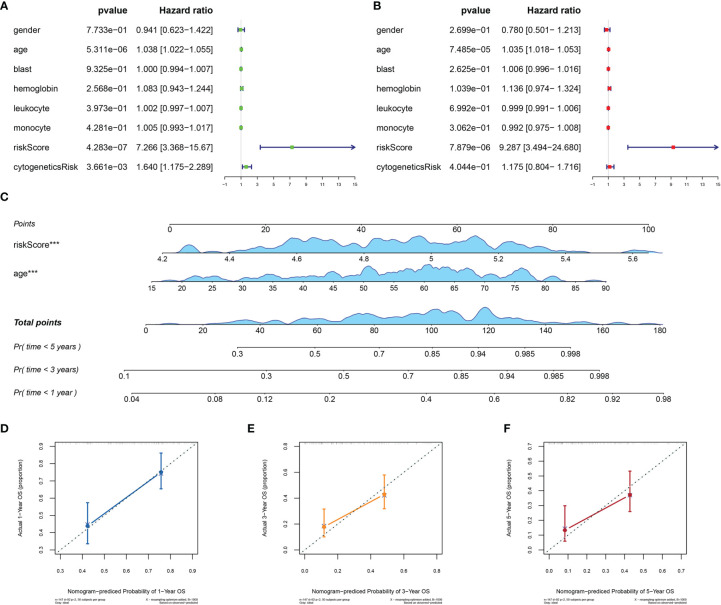
Efficiency of the MRG risk signature in prognostic prediction in AML from TCGA database. **(A, B)** Univariate and multivariate Cox analyses, which evaluated the risk signature’s independence in prognostic value in terms of overall survival in pediatric AML patients. **(C)** Nomograms for the probability of death at 1, 3, and 5 years. **(D–F)** The calibration curve of the nomograms. MRG, mitochondria-related gene; AML, acute myeloid leukemia; TCGA, The Cancer Genome Atlas. ***p < 0.001 in multivariate Cox analysis.

To assess the independence of MRG signature in clinical application, we performed univariate and multivariate Cox regression analyses in TCGA-AML dataset. The risk scores and clinicopathological characteristics, including age, gender, bone marrow blast cell, peripheral leukocyte, peripheral monocyte, hemoglobin, and cytogenetics risk category, were used as covariates. The univariate and multivariate Cox regression analyses revealed that both age and risk score were independent prognostic factors of OS, and risk score is superior to age (**Figures 5A, B**). These results indicated that the prognostic signature could be an independent unfavorable prognostic model for AML patients. With the use of multivariable Cox regression analysis, a nomogram (1, 3, and 5 years) was established to visualize the MRG risk model (**Figure 5C**). The corresponding calibration line for the nomogram showed good precise prediction (**Figures 5D** **–F**).

### Identification of Mitochondria-Related Gene−Related Signaling Pathways With Gene Set Enrichment Analysis

**Figure 6:**
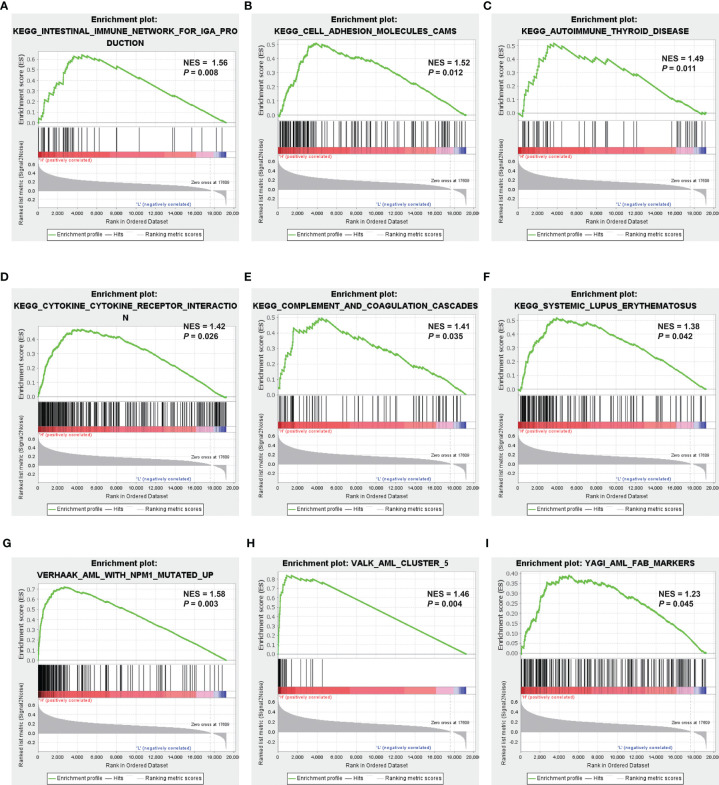
Identification of MRG-related signaling pathways with GSEA. **(A–F)** GSEA results of the gene KEGG enrichment in the AML patients from TCGA. **(G–I)** GSEA results of AML-related gene sets enrichment by the AML-TCGA data. MRG, mitochondria-related gene; GSEA, gene set enrichment analysis; KEGG, Kyoto Encyclopedia of Genes and Genomes; AML, acute myeloid leukemia; TCGA, The Cancer Genome Atlas.

GSEA was applied to compare the two MRG risk groups to explore which signaling mechanisms were triggered in the high-risk group, showing that higher-risk groups were found enriched in signaling molecule interaction, immune system, and immune diseases, such as cytokine–cytokine receptor interaction, cell adhesion, intestinal immune network for IGA production, autoimmune thyroid disease, and systemic lupus erythematosus (**Figures 6A** **–F**). Besides, as shown in **Figures 6G–I**, several AML-related gene sets from C2 curated gene sets in MSigDB were also enriched in the high-risk groups, which included Verhaak’s AML with NPM1 mutated upregulation, Valk’s AML cluster 5, and Yagi’s AML FAB markers. Therefore, the above results suggested that MRG−related classification is highly related to immune response and AML progress.

### Immune Landscape Between High and Low Mitochondria-Related Gene-Related Risk Groups of Acute Myeloid Leukemia Patients

**Figure 7:**
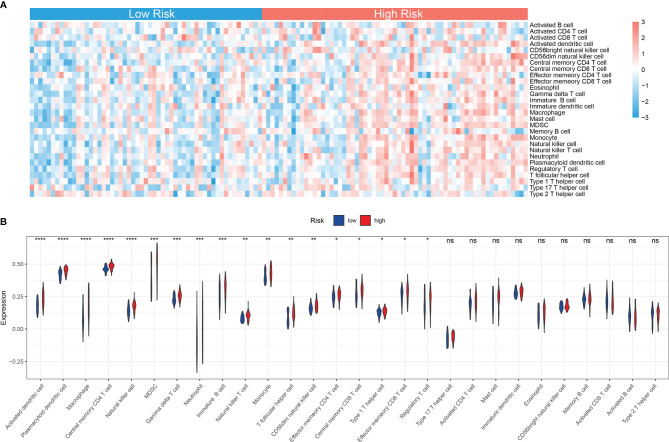
Immune landscape between low and high MRG risk groups of AML patients. **(A)** Heatmap of immune infiltration of 28 immune cell types between two MRG risk groups in TCGA-AML cohort. **(B)** Box plots showing significant differences of immune cells between two MRG risk groups. MRG, mitochondria-related gene; AML, acute myeloid leukemia. *p < 0.05, **p < 0.01, ***p < 0.001 and ns, not significant, high risk group versus low risk group.

Accumulating studies have shown that the mitochondria-related biological process such as mitophagy could protect tumor cells from antitumor immune responses, therefore promoting immune escape (26, 27). Also, the GSEA showed that many immune-related pathways were enriched in the high-risk group (**Figure 6A**). So the function of the MRG risk classification in the immune landscape was further explored. Thus, we estimated the differences in the 28 immune cell types’ immune penetration between low- and high-risk AML patients using the ssGSEA algorithm. The results of the immune landscape of AML patients are summarized in **Figure 7A**, showing that AML patients with high MRG risk had significantly higher proportions in memory CD4+ T cell, neutrophils, macrophages, monocyte, dendritic cell, natural killer (NK) cells, myeloid-derived suppressor cell (MDSCs), regulatory T cells, and immature B cells. However, there was no significant difference between the 2 groups in the activated CD4+, CD8+ T cell, CD56 bright NK cell, and activated B cell (**Figure 7B**). Therefore, MRG classification might be highly related to an immunosuppressive microenvironment.

### Immunosuppressive Microenvironment in High Mitochondria-Related Gene Risk Group

**Figure 8:**
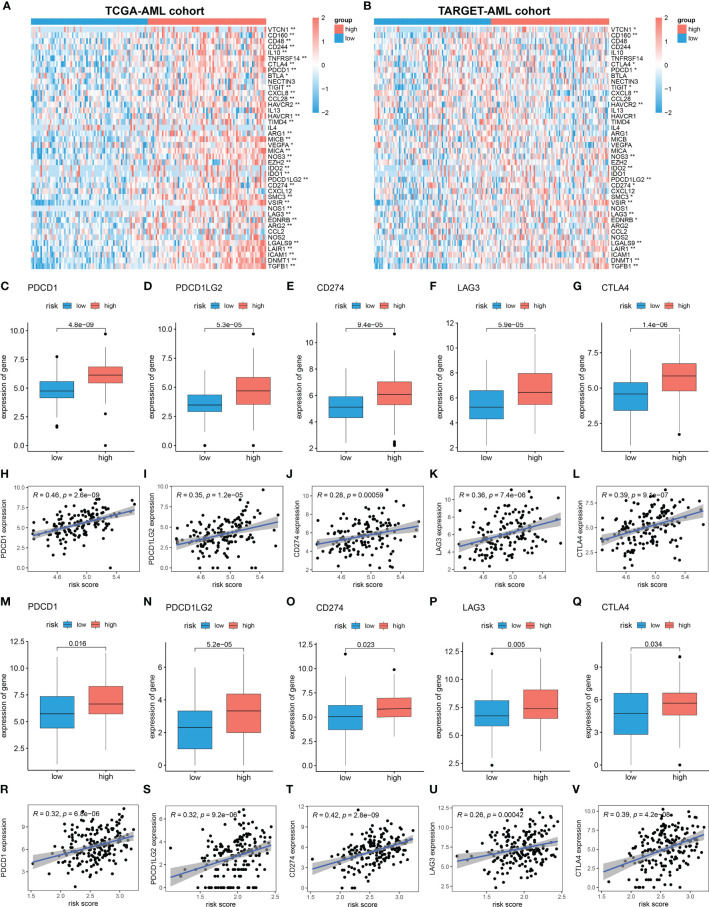
Immunosuppressive microenvironment in high MRG risk group. **(A, B)** Heatmaps of the immunosuppressive genes in high and low MRG risk groups in TCGA **(A)** and TARGET **(B)**. **(C–L)** Expressions of 5 immune checkpoints (PD1(PDCD1), PDL1(CD274), PDL2(PDCD1LG2), LAG-3, and CTLA-4) in two MRG risk groups and their correlation with MRG risk score in AML-TCGA patients. **(M–V)** Expressions of 5 immune checkpoints (PD1(PDCD1), PDL1(CD274), PDL2(PDCD1LG2), LAG-3, and CTLA-4) in two MRG risk groups and their correlation with MRG risk score in AML-TARGET patients. MRG, mitochondria-related gene; TCGA, The Cancer Genome Atlas. *p < 0.05 and **p < 0.01, high risk group versus low risk group.

Cancer-Immunity Cycle manages the delicate balance between the recognition of cancer and the prevention of autoimmunity. Immune escape of cancer cells is largely achieved by disrupting certain steps in the tumor immune cycle (28, 29). This cycle is often suppressed by several genes, which could induce the immunosuppressive microenvironment of cancer (30). Thus, in this study, 42 immunosuppressive genes involved in seven-step anticancer immunity were obtained from the Tracking Tumor Immunophenotype database (TIP; http://biocc.hrbmu.edu.cn/TIP/index.jsp) (31). Then the expression level of immunosuppressive genes in high and low MRG risk groups was explored. As shown in the heatmaps, most of these immunosuppressive genes were found significantly upregulated in the high MRG risk group in both TCGA and TARGET AML cohorts (**Figures 8A, B**). Immune checkpoint genes are essential for immune escape and immunotherapy of AML (32, 33). In this study, 5 common immune checkpoints genes, including PD1(PDCD1), PDL1(CD274), PDL2(PDCD1LG2), LAG3, and CTLA4, were found to be significantly upregulated in the high MRG risk group and positively associated with MRG risk score in both TCGA cohort and TARGET cohort (**Figures 8C** **–V**). Moreover, other than immune checkpoint genes, 12 immunosuppressive genes including VTCN1, CD160, TIGIT, NOS3, IDO2, SMC3, VSIR, EDNRB, LGALS9, LAIR1, DNMT1, and TGFB1 were found significantly higher in the high-risk groups in both TCGA cohort and TARGET cohort (**Supplementary Figure 3**).

## Discussion

More and more evidence suggests that a mitochondrion plays a key role in regulating cell energy level, apoptosis, and metabolism, which in turn could influence cell proliferation and differentiation, leading to the accumulation of immature myeloid progenitor in the hematopoietic system, which is the major manifestation of AML (34, 35). Studies have also demonstrated the clinical significance of mitochondrial targets for their effectiveness against relapsed or refractory AML (11, 23, 35). Thus, the identification of mitochondria-related prognostic biomarkers can be used to predict the prognosis of AML to improve patient management.

In this study, for the first time, a prognostic model based on 4 MRGs was constructed and validated for AML patients. The model performed well in predicting the OS state of AML patients in TCGA training and TARGET validation cohorts. Furthermore, the prediction efficacy of the risk model was superior to that of bone marrow blasts, leukocyte level, and cytogenetic risk, which are previously reported to be popular risk factors for AML development (24). Additionally, the correlation analysis has shown that the MRG-based risk score and expression level of 4 MRGs in the classifier were positively related to the cytogenetic risk of samples, showing a good prediction efficacy on AML prognosis. All 4 MRGs of the risk model, ETFB, CPT1A, HPDL, and IDH3A, were risk-associated and highly expressed in the high-risk group, indicating potential roles of these genes in the development of AML. Among them, HPDL, 4-hydroxyphenylpyruvate dioxygenase-like protein, a previously uncharacterized protein, localized in mitochondria, where it may function as 4-hydroxyphenylpyruvate dioxygenase, which was recently reported to be positively associated with the development of pancreatic ductal adenocarcinoma (PDAC) (36), AML (37), and breast cancer (38). Overexpression of HPDL promotes tumorigenesis and protects tumor cells from oxidative stress by reprogramming the metabolic profile of PDAC cells toward glutamine metabolism (36). ETFB, electron transfer flavoprotein subunit beta, is located in the inner membrane of the mitochondrial matrix in a complex with ETFA, FAD, and AMP, which together function as an electron acceptor in the fatty acid oxidation cascade and subsequent ATP production (39). However, recent studies have reported ETFB as a novel prognostic biomarker of many cancers, such as follicular carcinoma and breast cancer (40, 41). CPT1A catalyzes the rate-limiting step of the fatty acid oxidation (FAO) pathway, promoting cell proliferation and suppressing apoptosis (42). Abnormal CPT1A expression was associated with the poor OS of AML (43), ovarian cancer (44), and glioblastoma stem cells (45). Leslimar et al. reported that CPT1A could regulate prostate cancer survival in hypoxic conditions and promote aggressiveness (46). IDH3A, isocitrate dehydrogenases 3 Catalytic Subunit Alpha, is the key part of isocitrate dehydrogenases, catalyzing the oxidative decarboxylation of isocitrate to α-ketoglutarate in citrate cycle (47). Recently, a study reported that IDH3A could regulate one-carbon metabolism in glioblastoma *via* the IDH3A-cSHMT signaling axis, promoting cancer progression through metabolic reprogramming (48). Abnormal IDH3A expression was associated with the poor OS of lung and breast cancer patients, promoting tumor growth by inducing HIF-1-mediated metabolic reprogramming and angiogenesis (49).

The GSEAs show that the enriched pathways in AML patients with higher MRG risk mainly related to signaling molecule interaction, immune system, immune diseases, such as cytokine–cytokine receptor interaction, cell adhesion, intestinal immune network for IGA production, autoimmune thyroid disease, and systemic lupus erythematosus (**Figure 6**). Moreover, several AML-related gene sets from C2 curated gene sets in MSigDB were also enriched in the high-risk groups including AML with Verhaak’s AML with NPM1 mutated upregulation, Valk’s AML cluster 5, and Yagi’s AML FAB markers, showing a close relationship with AML prognosis. Mitochondria deeply involved in energy generation, differentiation, and activation processes of immune cells play a key role in the immune system, regulating innate and adaptive immunity (50–52). Mitochondrial dysfunction is also involved in many immunological diseases, such as systemic lupus erythematosus (53), rheumatoid arthritis (54), and type 1 diabetes (55).

Accumulating evidence shows that immune cells are closely related to the tumor microenvironment (TME) (56–58). It was reported that the innate immune cells (macrophages, neutrophils, dendritic cells, innate lymphoid cells, MDSCs, and NK cells) as well as adaptive immune cells (T cells and B cells) could promote tumor progression in TME (58). Moreover, mitochondria-related metabolic reprogramming in cancer cells deeply affects gene expression, cellular differentiation, and the TME (59). In our research, we found that AML patients with high MRG risk had significantly higher proportions in memory CD4+ T cell, neutrophils, macrophages, monocyte, dendritic cell, NK cells, MDSCs, regulatory T cells, and immature B cells. However, there was no significant difference in the activated CD4+, CD8+ T cell, CD56 bright NK cell, and activated B cell, suggesting that MRG classification might be highly related to an immunosuppressive microenvironment. It was reported that tumor-associated macrophages can account for up to 50% of some tumor mass, supporting tumor progression and resistance to drugs by providing cancer cell nutritional support (60). Tumor-associated macrophages and neutrophils were reported to be protumoral, promoting tumor cell invasion and metastasis and angiogenesis, remodeling extracellular matrix, and suppressing immune surveillance (61). Dendritic cells have been reported to be tumor-promoting in TMEs and to correlate with a positive prognosis in endometrial carcinoma (62). NK cells are considered killers of tumor cells. However, their activity is often suppressed in the TME, due to nutrient and oxygen deprivation, tumor-derived metabolic end products, and impaired metabolism in the TME (63). MDSCs are one of the major players in the TME, exerting immune-suppressive activity (64). Regulatory T cells could also suppress anticancer immunity and inhibit effective antitumor immune responses in TME (65). Immune checkpoints play a crucial role in carcinogenesis and development for enhancing immunosuppression in cancer (66). Ok et al. pointed out that in hematological malignancies, the common targets of immune checkpoints mainly include PD1, PD-L1, PD-L2, CTLA-4, TIM-3, and LAG3 (67). In our study, 5 common immune checkpoints (PD1, PDL1/2, LAG3, and CTLA4) were significantly upregulated in the high MRG risk group and positively related to MRG risk scores, suggesting an immunosuppressive bone marrow microenvironment in the high MRG risk group (**Figure 8**). It was reported that there is resistance to immunotherapy in most leukemia patients, partially due to the immunosuppressive bone marrow microenvironment (68). Moreover, leukemia cells could create the immunosuppressive bone marrow microenvironment through reprogramming metabolism to generate enough energy and to escape antitumor immune surveillance (68). Additionally, leukemia cells escape immune recognition through expressing inhibitors or immune checkpoint molecules such as PD-L1 or CTLA-4 (69).

Inevitably, due to the limitations of data sources and research methods, the present study has some deficiencies. Firstly, it was a retrospective study based on public databases of TCGA, GTEx, and TARGET, consisting of only 673 samples included, and this still requires further verification *in vivo* and *in vitro*. Finally, the clinical application of the MRG-based model still requires large-scale, multicenter, and in-depth research.

In conclusion, this was the first research to establish a mitochondria-related prognostic model for AML, which could be used as an independent prognostic indicator for AML patients. Moreover, we also found that high MRG risk AML patients were closely associated with an immunosuppressive microenvironment, indicating that attenuating immunosuppression in the bone marrow microenvironment may be an important treatment for AML. Additionally, we identified several targeted therapy drugs for MRG risk signature. Our study may provide a reference for the clinical prognosis and treatment of AML based on the regulation of mitochondrial function.

## Data Availability Statement

The datasets presented in this study can be found in online repositories. The names of the repositories can be found in the data acquisition section.

## Author Contributions

NJ, XZ, JW and BL designed the research study. NJ, QC and SW carried out the data analysis. NJ and FK prepared the manuscript. JL, HL, and JZ revised the manuscript critically. BL and JW did the final approval of the version to be published. All authors contributed to the article and approved the submitted version.

## Funding

This research was funded by grants from the National Natural Science Foundation of China [grant numbers 81774013, 82074129 and 31771534], the National Major Science and Technology Project of the Ministry of Science and Technology of China [grant number 2018ZX09721004-006-004], the Science and Technology Planning Project of Sichuan Province, China [grant numbers 2019JDPT0010, 2019YJ0473, and 19PTDJ0026], and Science and Technology Program of Luzhou, China [grant numbers 2019LZXNYDJ05, 2020LZXNYDJ30, 2018LZXNYD-PT02, 2020LZXNYDZ03, 2020LZXNYDP01, and 2018LZXNYD-YL05].

## Conflict of Interest

The authors declare that the research was conducted in the absence of any commercial or financial relationships that could be construed as a potential conflict of interest.

## Publisher’s Note

All claims expressed in this article are solely those of the authors and do not necessarily represent those of their affiliated organizations, or those of the publisher, the editors and the reviewers. Any product that may be evaluated in this article, or claim that may be made by its manufacturer, is not guaranteed or endorsed by the publisher.

## Supplementary Material

Supplementary Figure 1 MRGs differential expression analysis between TCGA-AML and GTEx are shown in the **(A–C)** Heatmap and **(D–F)** volcano plot.

Supplementary Figure 2 High expression level of the 4 MRGs signature in high MRG risk group.

Supplementary Figure 3 Immunosuppressive genes promoted in high MRG risk group in **(A–L)** TCGA-AML cohort and **(M–X)** TARGET cohort.

Supplementary Table 1 MRGs list from MitoCarta3.0.

Supplementary Table 2 Differential expression list of MRGs.

Supplementary Table 3 Unicox analyisis of Differentially expressed MRGs (p<0.05).
