# Supplementary figures and images for: Identification of a Mitochondria-Related Gene Signature to Predict the Prognosis in AML
Source: Front Oncol. 2022 Mar 10;12:823831. doi: 10.3389/fonc.2022.823831 (PMC8960857; doi:10.3389/fonc.2022.823831)

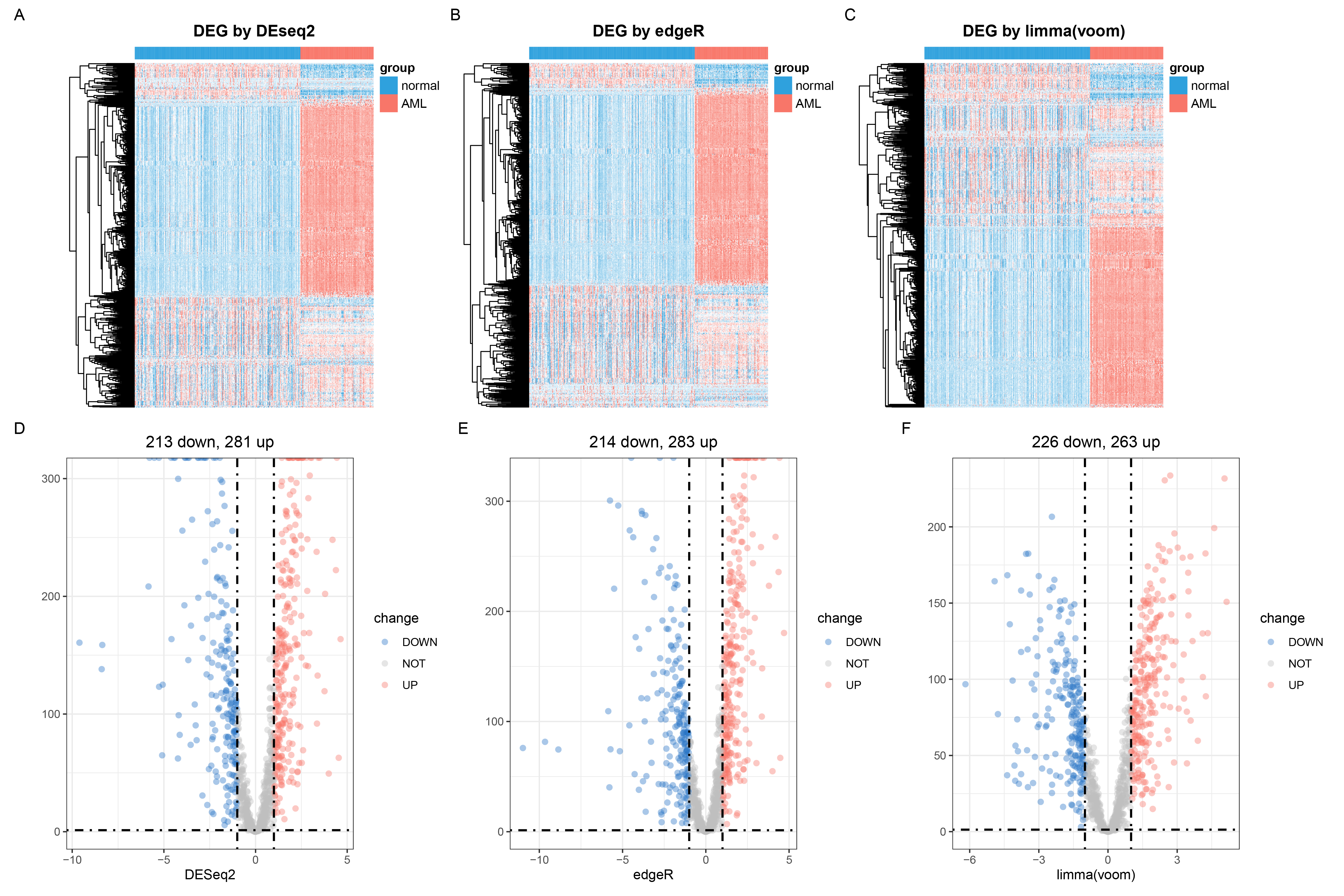

Supplement: Supplementary Figure 1 — MRGs differential expression analysis between TCGA-AML and GTEx are shown in the (A–C) Heatmap and (D–F) volcano plot. [file Image_1.jpeg]

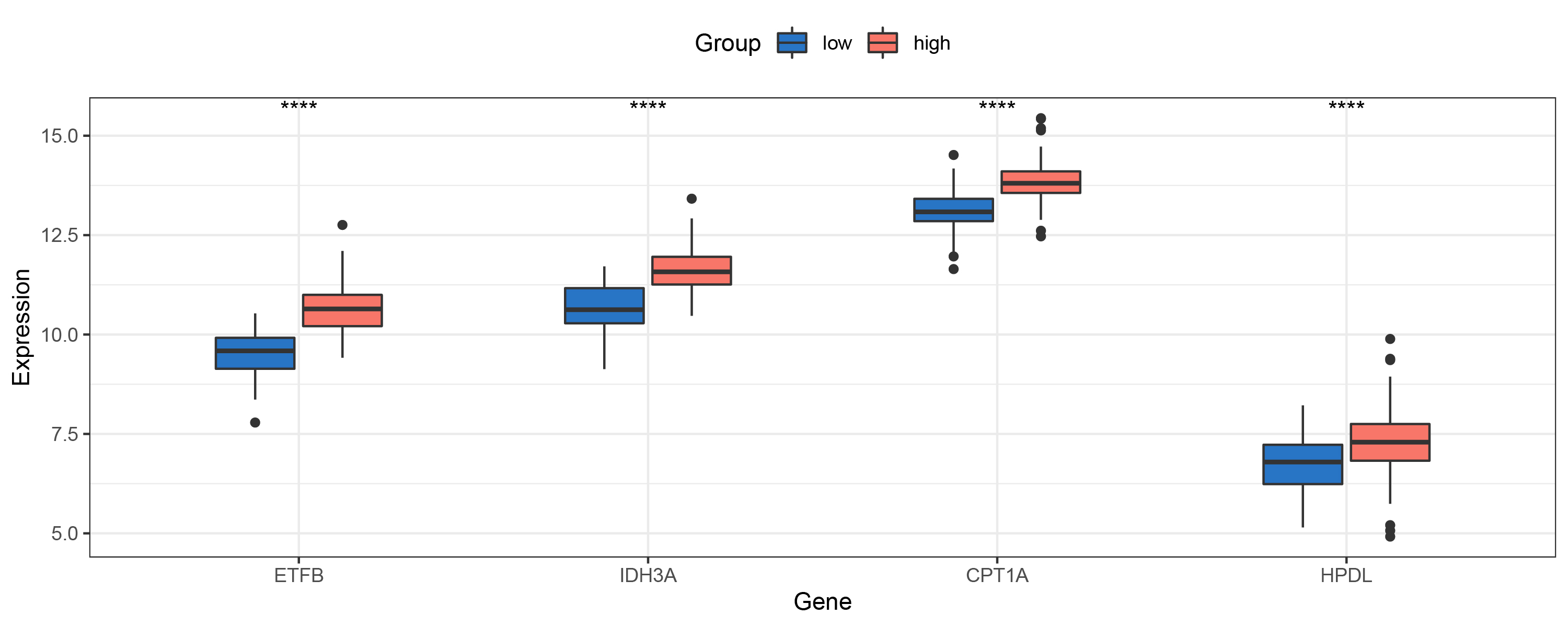

Supplement: Supplementary Figure 2 — High expression level of the 4 MRGs signature in high MRG risk group. [file Image_2.jpeg]

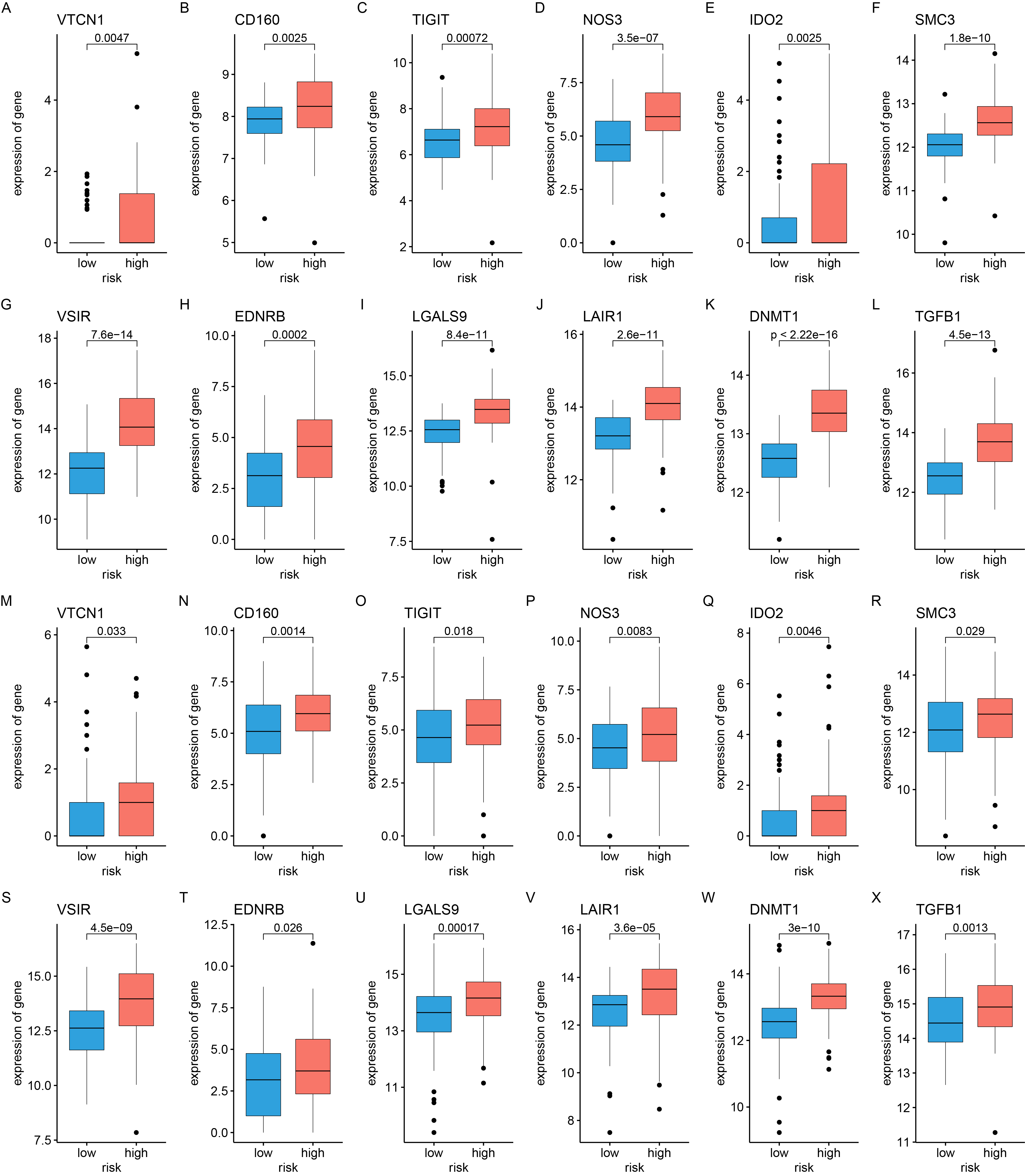

Supplement: Supplementary Figure 3 — Immunosuppressive genes promoted in high MRG risk group in (A–L) TCGA-AML cohort and (M–X) TARGET cohort. [file Image_3.jpeg]
